# Curcumin as an Antiviral Agent

**DOI:** 10.3390/v12111242

**Published:** 2020-10-31

**Authors:** Morgan R. Jennings, Robin J. Parks

**Affiliations:** 1 Regenerative Medicine Program, Ottawa Hospital Research Institute, Ottawa, ON K1H 8L6, Canada; mojennings@toh.ca; 2 Department of Biochemistry, Microbiology and Immunology, University of Ottawa, Ottawa, ON K1N 6N5, Canada; 3 Centre for Neuromuscular Disease, University of Ottawa, Ottawa, ON K1N 6N5, Canada; 4 Department of Medicine, The Ottawa Hospital, Ottawa, ON K1H 8L6, Canada

**Keywords:** curcumin, antiviral, broad-spectrum, phytochemical

## Abstract

Curcumin, the primary curcuminoid compound found in turmeric spice, has shown broad activity as an antimicrobial agent, limiting the replication of many different fungi, bacteria and viruses. In this review, we summarize recent studies supporting the development of curcumin and its derivatives as broad-spectrum antiviral agents.

## 1. Introduction

Curcumin (diferuloylmethane) is the primary curcuminoid derived from the rhizome of *Curcuma longa* plant [1,2], and is typically used both as a strong food dye and consumed as a spice in the form of turmeric [1]. In addition, curcumin has seen wide use in traditional medicine throughout Asia, due to its anti-inflammatory and wound-healing properties [3,4]. Modern research has also demonstrated that curcumin has diverse biological functions, with reported anti-cancer, antioxidant and anti-microbial properties [3,5,6]. Curcumin can act not only as an anti-fungal and anti-bacterial compound, but also as an anti-viral compound, inhibiting replication in a wide-range of viruses [7], as summarized in Table 1. In this review, recent research into the antiviral properties of curcumin, its derivatives, and formulations will be discussed.

## 2. Curcumin

**Figure 1:**
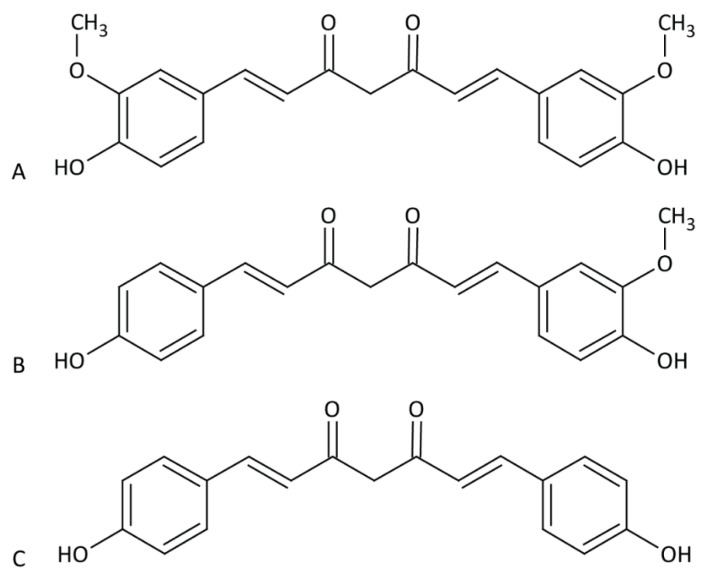
Chemical structure of curcuminoids. Chemical structure of curcumin (**A**), demethoxycurcumin (**B**) and bisdemethoxycurcumin (**C**).

Approximately 3–5% of ground and dried turmeric are the curcuminoids [27]. The curcuminoids are comprised of curcumin (77%), demethoxycurcumin (17%) and bisdemethoxycurcumin (6%) (Figure 1). For the purpose of this review, we will refer to the purified products by name, and curcuminoid will refer to two or more of these compounds together. Curcumin is “generally recognized as safe” by the Food and Drug Administration (FDA) [28], with consumption of doses up to 5 g/kg reported to have no toxic effects in rats [29]. For comparison, the median lethal dose (LD_50_) of table salt in rats is approximately 3 g/kg [30].

The first suggestion that curcumin had antiviral properties came in the 1990s with the discovery that curcumin could inhibit the human immunodeficiency virus (HIV) viral protease in vitro, with a median inhibitory concentration (IC_50_) of 100 µM [31]. Since then, numerous studies have reported on the antiviral properties of curcumin against a diverse set of viruses, including those with RNA and DNA genomes, both enveloped and non-enveloped, as discussed in greater detail below.

Despite promise as an antimicrobial agent, curcumin has several limitations. Curcumin is practically insoluble in water, with low in vivo bioavailability. As little as 1% of administered curcumin is absorbed by the body, and it remains virtually undetectable in target tissues [32,33]. For example, ingestion of 12 g of curcumin resulted in a concentration of 29.7 ng/mL (~81 nM) of curcumin in human blood serum one-hour (h) post-ingestion [34], which is far below any effective concentration reported in many in vitro studies. For example, curcumin is needed at an IC_50_ of 40 µM to inhibit the HIV-1 integrase [35], while the effective dose against influenza A virus (IAV) is ~10 µM [36]. In addition, curcumin is unstable at physiological pH, with a half-life in growth medium containing serum of approximately 8 h, and rapidly degrades into several ineffective products [1]. Such limitations have prevented translation of the many very promising in vitro findings with curcumin into clinical benefit.

To overcome these obstacles, researchers have explored the use of curcumin derivatives. These compounds include the other curcuminoids present in turmeric besides curcumin, as well as synthetic curcumin analogues. Typically, these compounds maintain a particular chemical moiety of curcumin that is responsible for biological efficacy, but are designed to improve biological activity, solubility and/or stability. One such example includes the synthesis of novel carbocyclic curcumin derivatives (addition of a carbon ring to one of curcumin’s ketone groups), which led to improved HIV-1 protease inhibition [37]. Many research groups have sought to improve delivery of curcumin through the use of various formulations such as nanoemulsions and nanoparticles. Examples include liposome-encapsulated curcumin, which improved stability in phosphate-buffered saline (PBS) and reduced toxicity compared to free curcumin [38], and curcumin-loaded apotransferrin nanoparticles, which improved cellular uptake [39]. Further examples of chemical derivatives and alternative formulations for delivery and stability are provided throughout this review.

## 3. RNA Viruses

### 3.1. Human Immunodeficiency Virus

Perhaps the largest body of work on the anti-viral properties of curcumin pertain to its efficacy against HIV. Indeed, curcumin can impact HIV function at several different stages of the virus lifecycle. Given the anti-inflammatory properties of curcumin, Ferreira et al. [10] evaluated whether curcumin could reduce inflammation in the female genital tract (FGT), which is known to facilitate HIV acquisition. Epithelial cells lining the FGT play a key role in forming a primary barrier to HIV entry, yet exposure of genital epithelial cells (GECs) to intact virus or HIV-1 glycoprotein 120 (gp120) induces an inflammatory response that results in downregulation of tight junction (TJ) proteins [40]. This loss of barrier integrity may allow HIV-1 to traverse the genital epithelium and establish an infection. Pre-treatment of primary GECs with 5 µM of curcumin prevented the down-regulation of TJ proteins, thus potentially reducing HIV infection rates [10]. Treatment of chronically infected T-cells (H9 T-cells) with at least 5 µM curcumin significantly reduced expression of p24, a marker of virus replication, at 24 hpi compared to untreated cells [10]. This inhibition could be maintained for several days if the curcumin in the medium was replaced every 24 h [10]. Curcumin can inhibit HIV replication through impacting the function of several viral proteins including the viral integrase, protease, as well as the trans-activator of transcription (Tat) protein [31,35,41]. Curcumin may inhibit the viral integrase through direct interaction with the catalytic core of the protein [35]. Similarly, computer modeling suggests that curcumin may also bind the active site of the HIV-1 protease [31,42]. In the case of Tat, treatment with curcumin induces Tat degradation in a dose- (20 to 120 µM) and time-dependent manner, which appears to be mediated through a proteasomal pathway independent of ubiquitination [26]. Five µM of curcumin can also inhibit HIV-LTR activation by inflammatory cytokines induced through infection of other STI, such as HSV-1, HSV-2 and *N. gonorrhoeae* [10,43].

Hypothesizing that the β-diketone moiety of curcumin may be responsible for its decomposition at physiological pH, Kumari et al. [11] evaluated the anti-HIV-1 properties of a synthetic curcumin analogue lacking the β-diketone moiety, termed curcumin A. Curcumin A exhibited improved stability relative to curcumin in PBS and in serum in vitro, but similar stability in complete tissue culture medium [11]. Using an HIV reporter virus encoding luciferase, curcumin A reduced luciferase activity to a similar extent as curcumin in human T-cell leukemia (CEM) cells (IC_50_ of 0.8 and 0.7 µM, respectively) and with greater potency in primary peripheral blood mononuclear cells (PMBCs) (IC_50_ of 2 and 12 µM, respectively). Although both curcumin and curcumin A could inhibit early reverse transcription of the virus, only curcumin A lowered late viral genome copy levels. At least part of the therapeutic efficacy of curcumin and curcumin A appears to be due to their ability to activate heme oxygenase-1 (HO-1), since activation of HO-1 by heme inhibits HIV-1 [44]. Curcumin A was moderately less toxic than curcumin to both cell lines (median cytotoxic concentration (CC_50_) of 2.4 vs 1.26 µM, and 35 vs. 22 µM, respectively), suggesting that curcumin A has an improved therapeutic potential compared to native curcumin [11].

Curcumin-stabilized silver nanoparticles (Cur-AgNP) have also been investigated for their efficacy against HIV [45]. Treatment of ACH-2 cells with Cur-AgNP was more effective at lowering HIV-LTR expression compared to curcumin alone [45]. However, Cur-AgNP did not completely abolish HIV-1 p24 expression, as the levels of this protein continued to increase throughout the course of infection, albeit with significantly reduced kinetics relative to vehicle-treated cells. Cur-AgNP also showed greater immunomodulatory effects than curcumin, lowering expression of TNF-α, IL-6, IL-1β, and NF-κB [45]. These results suggest that stabilized nanoparticles may hold promise for improving the efficacy of curcumin for treating HIV, and possibly other viruses.

Mirani et al. [46] studied the efficacy of tetrahydrocurcumin, a colourless metabolite of curcumin, either alone or formulated as a microemulsion (designated TME), as a potential vaginal microbicide for topical application as a prophylactic approach to prevent HIV infection. In a reporter cell line, TME showed greater HIV-1 inhibition than the drug alone (IC_50_ of 0.9357 μM and 3.639 μM, respectively). In a p24 antigen assay, 1 µM of TME also showed a greater ability to lower p24 levels over a 10-day time course of infection compared to 5 µM of tetrahydrocurcumin [46]. To further validate the potential of TME as a vaginal microbicide, the effect of TME on *Lactobacillus* species was evaluated, as these bacteria form part of the natural microbiota and are important for maintaining the acidic environment of the FGT [47]. No change in viability was observed at any concentration for *L. casie* and *L. acidophilus* [46]. Finally, the authors showed that both TME and tetrahydrocurcumin were efficacious when formulated into suspension gels, which would be easy to apply, with no loss of anti-HIV-1 efficacy. Collectively, these studies show curcumin’s potential against HIV-1, both through direct inhibition and as a prophylactic strategy.

### 3.2. Zika Virus

Mounce et al. [14] compared the efficacy of curcumin and several curcumin analogues against Zika (ZIKV), chikungunya virus (CHIKV) and vesicular stomatitis virus (VSV), all enveloped viruses, as well as Coxsackie B3 virus (CVB3), a non-enveloped virus. Direct incubation of ZIKV in 10 µM to 1 mM of curcumin, bisdemethoxycurcumin, demethoxycurcumin or the synthetic curcumin structural analogue EF-24 and derivative FLLL31 (originally synthesized by Adams et al. [48] and Lin et al. [49], respectively), resulted in a dose- and time-dependent decrease in subsequent virus infection, suggesting these drugs have a direct ability to inactivate virus or inhibit cell attachment. EF-24 and FLLL31 were less effective than the curcuminoids, and only the curcuminoids had a lower IC_50_ relative to their CC_50_ in HeLa cells [14]. In several time-of-addition assays, only cells treated prior to or during infection reduced ZIKV recovery, indicating that curcumin acts against ZIKV exclusively during cell-attachment or entry and not at later stages of infection [14]. Incubation with curcumin was shown to reduce virus binding to the cell, but it did not lower the total number of virus particles, indicating that curcumin and its analogues are not viricidal compounds per se but rather prevent ZIKV attachment to cells. The authors speculated that, since curcumin has been reported to alter the membrane fluidity of the hepatitis C virus (HCV) envelope [50], it may work by a similar mechanism to prevent ZIKV cell attachment.

Gao et al. [15] evaluated a natural product library for phytochemicals, including curcumin, for compounds with anti-ZIKV activity. In their initial screen in Vero E6 cells, curcumin (amongst other compounds) had a lower IC_50_ (~5–14 µM) relative to CC_50_ (~53 µM) against several strains of ZIKV as determined by plaque assay. Time-of-addition assays again suggested that curcumin exerts anti-ZIKV activity through an ability to interfere with cell attachment [15]. Taken together, these studies demonstrate curcumin’s potential against ZIKV.

### 3.3. Dengue Virus

In addition to evaluating curcumin against ZIKV, Gao et al. [15] also investigated the same selection of compounds against dengue virus (DENV). Curcumin reduced plaque formation of all four strains (DENV-1-4, IC_50_ of 9.37, 3.07, 2.09, and 4.83 µM, respectively) examined in LLC-MK2 cells while showing limited toxicity (CC_50_ of 59.42 µM) [15]. Though the mechanism of inhibition was not addressed, an earlier study demonstrated that curcumin likely inhibits DENV-2 indirectly through impact on cellular systems rather than directly on viral functions [51].

Balasubramanian et al. [8] evaluated the anti-DENV properties of curcumin, bisdemethoxycurcumin and three other synthesized analogues. In an in vitro activity assay, curcumin and the four analogues modestly inhibited viral protease activity (IC_50_ of ~36–66 µM). Similarly, these compounds only modestly inhibited replication of a DENV2 reporter replicon construct, with the acyclic and cyclohexanone analogues of curcumin performing slightly better than the natural curcuminoids (50% effective concentration (EC_50_) of 8.61 and 8.07 µM versus 13.91 µM) [8]. However, the compounds showed greater activity in a plaque assay, with all analogues demonstrating greater inhibition than curcumin (EC_50_ of 2.34–6.49 µM versus 13.95 µM, respectively). The mechanism by which curcumin inhibits DENV appeared to be due to effects on cellular lipid metabolism. Curcumin and its derivatives downregulated acetyl-CoA carboxylase and fatty acid synthase, and lowered lipid droplet (LD) formation, processes that normally may function to enhance DENV infection [8]. Curcumin treatment also led to actin filament disorganization and defects in polymerization, another process that is naturally important for DENV entry and replication [8].

### 3.4. Chikungunya Virus

Pre-incubation of lentiviral vectors pseudotyped with chikungunya virus (CHIKV) envelop proteins E2 and E1 with curcumin prevented infection of HEK 293T cells (IC_50_ of 10.79 µM) [16]. Time-of-addition assays with CHIKV encoding an mCherry-tagged viral replicase showed that treatment with curcumin lowered the number of mCherry positive cells when added up to 2 hpi, but it had no effect thereafter. Immunofluorescence analysis revealed that curcumin treatment did not alter the intensity or pattern of mCherry staining within the cells, but it did reduce the total amount of cells positive for mCherry. Taken together, this study suggests that, much like ZIKV, curcumin prevents cell-entry or attachment, but it has no effect on virus replication [16]. A similar conclusion was obtained by Mounce et al. [14], who postulated that curcumin may be altering the conformation of viral surface glycoproteins, thus preventing viral attachment. Thus, for CHIKV, curcumin may be an effective treatment to prevent initial infection but is likely ineffective at controlling a pre-existing infection.

### 3.5. Vesicular Stomatitis Virus

As in the case with ZIKV and CHIKV, VSV infection was inhibited when the virus was incubated directly with curcumin (IC_50_ of 4.5 µM) [14,16]. Although these studies did not address the mechanism by which curcumin inhibited VSV, this effect can likely be attributed to inhibition of cell attachment [14].

### 3.6. Influenza A Virus

Curcumin is also a potent inhibitor of IAV, and likely exerts its effect at multiple different stages of the virus lifecycle. Incubation of IAV with curcumin results in reduced infectivity, possibly due to the ability of curcumin to interfere with viral haemagglutinin activity [19,36]. Curcumin also inhibits NF-κB signalling, which is required for IAV replication [52]. Time-of-addition experiments showed that addition of curcumin as late as 5 hpi reduced plaque formation (EC_50_ of ~58 µM), suggesting curcumin interferes with an early stage of virus gene expression or replication [19]. Additionally, curcumin inhibited several IAV-induced toll-like receptor (TLR) signalling pathways and proteins, which are normally required for efficient virus internalization and/or replication, including TLR2/4/7, MyD88, TRIF, and TRAF6. Indeed, treatment of cells with agonists for TLR2/4, p38/JNK MAPK or NF-κB were able to circumvent the replication block imposed by curcumin [19]. Importantly, curcumin treatment by oral gavage (50 and 150 mg/kg) reduced IAV replication and lung injury in an in vivo animal model [19], clearly illustrating that curcumin can provide a therapeutic benefit to combat infection and virus-induced disease. This latter observation was supported by a study by Han et al. [53], who demonstrated that mice infected with the IAV strain PR8 and fed 30 or 100 mg/kg of curcumin had increased survival, reduced bodyweight loss, and lower IAV burden in lung tissues as determined by immunohistochemistry. Bronchoalveolar lavage (BAL) fluid and lung tissues from infected, curcumin-treated mice had lower levels of monocyte chemoattractant protein-1 (MCP-1) and tumour necrosis factor-alpha (TNFα) compared to untreated mice, suggesting reduced inflammation. Similarly, bone marrow-derived macrophages (BMDM cells) isolated from mice and infected with PR8 produced lower levels of inflammatory cytokines IL-6, TNF-α and MCP-1 following treatment with curcumin [53]. These results indicate that curcumin is not only capable of inhibiting IAV replication, but also attenuates IAV-induced lung disease, likely through inhibition of NF-κB signaling leading to reduced secretion of inflammatory cytokines by resident macrophages [53].

The enone functional groups (α,β-unsaturated carbonyls) of curcumin appear to be at least partially responsible for the anti-IAV properties of curcumin [54]. These enone groups can form Michael adducts with sulfhydryl (SH) groups on other proteins, suggesting that curcumin may exert anti-IAV activity through the ability to conjugate with viral surface protein(s) and interfere with their function [54]. Richart et al. [24] compared the effectiveness of curcumin and monoacetylcurcumin (MAC), a curcumin analogue that maintains the enone groups and shows improved stability. As with curcumin, direct incubation of IAV with MAC inhibited subsequent plaque formation (IC_50_ of ~0.2 µM), and 30 µM of MAC was more effective in limiting virus replication in long-term cultures (up to 36 h, [24]). Addition of GSH, which can act as a competitive inhibitor during the Michael addition reaction, to MAC reduced its ability to prevent plaque formation, once again highlighting the importance of the enone groups on MAC efficacy. Although both MAC and curcumin were capable of mildly reducing IAV neuraminidase (NA) activity (which is vital for viral egress) at high drug concentrations (200 µM), only MAC was effective at lower concentrations (25 µM) [24]. However, unlike curcumin, MAC was unable to reduce haemagglutinin (HA) activity at any concentration tested, indicating MAC cannot prevent cell attachment [24]. Compared to curcumin, MAC was a stronger suppressor of the PI3K/Akt signalling pathway, which is required for IAV membrane fusion, suggesting at least part of the anti-IAV efficacy of MAC may be due to its effects on membrane fusion and virus internalization. Given that curcumin and MAC appear to inhibit IAV via different mechanisms, the authors also showed that combined treatment with both compounds was more effective than either compound alone.

Combining several aspects of the studies described above, Lai et al. [55] analyzed curcumin and several analogues for anti-IAV activity. In MDCK cells, treatment with the maximum non-toxic dose of each compound significantly reduced mRNA levels of the IAV M gene in infected cells, with curcumin showing the greatest reduction. Using immunohistochemistry, only curcumin and tetramethylcurcumin inhibited the nuclear export of the IAV nucleoprotein, thus preventing viral assembly. All compounds reduced neuraminidase activity. Consistent with the previous studies, in vivo treatment of mice with 25–100 mg/kg of curcumin reduced lung pathology compared to untreated controls [55]. Additionally, both pre- and post-infection treatment with curcumin improved average survival time of infected mice [55].

Taken together, treatment with curcumin or curcumin analogues can inhibit IAV through several means, including preventing entry [24,55], inhibiting replication [19,55], and preventing exit [24,55]. Additionally, oral treatment with curcumin improved the survival of IAV-infected mice [19,53,55], indicating the potential of curcumin and its analogues against IAV infection.

### 3.7. Enterovirus 71

Though previous research has demonstrated that curcumin can inhibit human enterovirus 71 (EV71) replication in vitro [56], those studies were performed using Vero cells, which are non-human kidney epithelial cells. To better approximate the in vivo conditions of EV71 infection, Huang et al. [20] evaluated curcumin against EV71 in HT29 human intestinal epithelial cells. Treatment with 10 µM of curcumin significantly reduced viral protein expression, genome replication and titer, and prevented EV71-induced cell death. Time-of-addition assays revealed that curcumin did not affect viral attachment and entry, but it effectively inhibited protein expression during early stages of infection [20]. These effects appear to be at least partly due to the ability of curcumin to inhibit protein kinase C-δ (PKCδ). Infection of a cell with EV71 induces phosphorylation of a key activating residue of PKCδ, Tyr311, which is reduced in curcumin-treated cells. Knockdown of PKCδ using siRNA or administration of the PKCδ inhibitor rottlerin also drastically reduced viral protein expression, indicating the importance of PKCδ activation for optimal EV71 gene expression [20]. Treatment with 10–20 µM of curcumin also reduced viral protein expression in C2BBe1 cells that had been differentiated into mature intestinal epithelial cells [20].

Lin et al. evaluated several curcumin-derived carbon quantum dot (Cur-CQD) formulations against EV71 [13]. These quantum dot formulations improve curcumin’s solubility in water, which may explain its increased antiviral activity [13]. In human rhabdomyosarcoma (RD) cells, all Cur-CQD formulations had superior CC_50_ and EC_50_ scores compared to curcumin, with Cur-CQD-180 (named for the heat applied during synthesis, i.e., 180 °C) having the highest selectivity index (SI, <0.07 vs. 2261.0, respectively) inhibiting EV71-induced cytopathic effect (CPE) and virus recovery [13]. Cur-CQD-180 lowered expression of several viral proteins when added post-infection, such as the structural protein VP1 and non-structural proteins 3CD^pro^ and 3D^pol^, in a dose-dependent manner [13]. Additionally, Cur-CQD-180 reduced the cleavage of eukaryotic translation initiation factor 4G (eIF4G), which the virus utilizes to inhibit host protein synthesis while encouraging its own [13,57]. In vivo, treatment of EV71-infected mice with Cur-CQD-180 improved survival, lowered pathology scores and prevented virus-induced weight loss compared to untreated and curcumin-treated mice [13]. Cur-CQD-180 treatment also reduced the quantity of viral mRNA and protein that were detected in brain and limb muscle tissue [13]. These results demonstrate the potential of curcumin formulations in bypassing the limitations of native curcumin for in vivo treatment against EV71.

### 3.8. Human Respiratory Syncytial Virus

Incubation of Human Respiratory Syncytial Virus (HRSV) with curcumin-stabilized silver nanoparticles (Cur-AgNP) produced particles of ~200 nm, which is larger than either Cur-AgNP or HRSV particles alone, suggesting Cur-AgNP binds directly to HRSV, and this binding appeared to inhibit virus infection [17]. Treatment of cells with Cur-AgNP post-infection was not as effective as the pre-treatment at reducing virus recovery [17]. A derivate of curcumin in which the drug was loaded into β-cyclodextrin and attached to sulfonated graphene oxide sheets (termed GSCC) was also evaluated against HRSV [18]. This formulation inhibited HRSV primarily by preventing viral attachment, but it also inhibited HRSV when applied to cells after infection [18]. Given the presence of sulfonic acid in the formulation and that HRSV can utilise heparin sulfate for entry [58,59], the authors suggest the GSCC sheets prevent infection by acting as a competitive inhibitor to sequester viruses away from the cell. Additionally, both pre- and post-treatment with the GSCC sheets reduced viral G protein expression, which is responsible for viral attachment, indicating the GSCC sheets also can directly affect viral gene expression [18]. Taken together, these results clearly show that encapsulation can be used to improve the efficacy of curcumin against HRSV.

### 3.9. Norovirus

For enveloped viruses, direct incubation of curcumin frequently reduces the ability of the virus to infect cells, which is thought to be due to the ability of curcumin to bind to and inhibit the action of surface glycoproteins on the virus [14,15,16]. Somewhat surprisingly, incubation of murine norovirus (MuNoV) with ~679 µM curcumin also significantly lowered plaque formation, despite the fact that noroviruses are non-enveloped [60]. This effect was time- and dose-dependent, suggesting the reduction in plaque formation was due to direct neutralization of viral particles as opposed to preventing infection [60]. However, in human norovirus (HuNoV) replicon-bearing HG23 cells, curcumin had no effect on HuNoV replication [60], suggesting curcumin only affects virus particle integrity and does not alter other aspects of the virus lifecycle.

Curcumin has also been investigated against norovirus in the context of a photodynamic therapy (PDT), in which the compound in question (termed the sensitizer) is “activated” by exposure to specific wavelengths of light, leading to the production of reactive oxygen species (ROS). Treatment with 5 µM of curcumin photodynamically activated (PDAC) by blue light radiation reduced MuNoV titers with greater effect than curcumin or blue light alone [21]. The morphology of the virions were examined using TEM, and samples treated with PDAC had visible viral debris suggesting PDAC treatment is capable of physically disrupting MuNoV particles [21]. For an in vivo test of this therapy, live oysters, which are known to bio-accumulate norovirus, were exposed to MuNoV and curcumin for 6 h, the intestines of the oysters were then removed and exposed to blue light. Titration of virus from these tissues showed that, while the viral titer was reduced in a dose-dependent manner, curcumin was less effective in oyster tissues compared to virus in solution, which was attributed to lower final concentration of curcumin in the oysters and possible absorption of light by oyster tissues, reducing the fraction of energy reaching the curcumin [21].

Randazzo et al. [22] also evaluated PDAC against norovirus, using MuNoV and feline calicivirus (FCV) as HuNoV surrogates. PDAC treatment using up to 13.6 µM of curcumin reduced the 50% tissue culture infective dose (TCID_50_) of both FCV and MuNoV, but appeared more effective against FCV [22]. Increasing the concentration of viruses reduced the effect of PDAC, which the authors attributed to the limited quantity of ROS produced being spread over a greater quantity of virus—each particle receives less damage from the available ROS [22]. The strategy of using PDAC against norovirus in oysters represents a potentially simple solution to combat norovirus accumulation, given curcumin is already viewed as safe for use in the food industry.

### 3.10. Viral Hemorrhagic Septicemia Virus

Viral Hemorrhagic Septicemia Virus (VHSV) affects numerous species of fish throughout the world, with mortality as high as 90% [61]. Pre-treatment of fathead minnow (FHM) cells with 0–240 µM curcumin reduced CPE and improved cell viability in a dose-dependent manner [9]. VHSV infection significantly increases ROS production compared to uninfected cells, and pre-treatment with curcumin lowered ROS production to baseline levels, which could have contributed to the lower CPE observed in the curcumin pre-treated cells [9]. Additionally, 120 µM curcumin reduced viral genome copy numbers within infected cells, indicating curcumin can inhibit virus replication [9]. Based on protein network analysis, the authors speculated that curcumin reduced VHSV infection by downregulating heat shock cognate 71 kDa protein (HSC71), which is naturally upregulated by PNV during early infection. Consistent with this mechanism, treatment with heat shock protein inhibitor KNK437 reduced virus-induced CPE and lowered viral genome copy numbers within the cell [9].

### 3.11. Porcine Reproductive and Respiratory Syndrome Virus

Porcine reproductive and respiratory syndrome virus (PRSSV) causes large economic losses in the swine industry [62]. Although a vaccine exists against PRSSV, adverse effects from vaccine treatments have led researchers to look for antiviral compounds [63]. Incubation of 15 µM of curcumin directly with several strains of PRRSV prevented infection of Marc-145 cells (permissive to PRRSV infection) [25]. While curcumin did not lower expression of surface receptors or prevent viral attachment, curcumin appeared to inhibit PRRSV cell membrane fusion and internalization. Similar results were obtained in primary porcine alveolar macrophage [25].

### 3.12. Transmissible Gastroenteritis Virus

Another virus that causes significant livestock and economic loss in the pork industry is transmissible gastroenteritis virus (TGV), a porcine coronavirus [64]. Noting curcumin’s activity against other enveloped viruses, Li et al. [65] evaluated curcumin against TGV. Direct incubation with at least 20 µM of curcumin prior to infection reduced viral yield. Furthermore, treatment with curcumin caused a dose-dependant decrease in viral absorption, and a reduction in both viral yield (IC_50_ of 8.6 µM) and protein levels at concentrations that are non-toxic to porcine kidney (PD-15) cells [65].

### 3.13. Severe Acute Respiratory Syndrome Coronavirus 2

While there have been no published research articles at the time of writing on any antiviral effect of curcumin on severe acute respiratory syndrome coronavirus 2 (SARS-CoV-2), the causative agent in the worldwide COVID-19 pandemic, it has been speculated that curcumin could inhibit SARS-CoV-2 replication. Curcumin has already been demonstrated to inhibit SARS-CoV-1 replication (EC_50_ of >10 µM), the coronavirus which caused the 2003 epidemic [66]. Additionally, several molecular docking studies have been performed that suggest curcumin would be effective at inhibiting SARS-CoV-2 replication [67], through interacting with the spike glycoprotein and inhibiting angiotensin-converting enzyme 2 (ACE2) [68], inhibiting the viral non-structural protein Nsp15 [69], or inhibiting the main viral protease [70].

## 4. DNA Viruses

### 4.1. Herpes Simplex Virus 2

Herpes Simplex Virus 2 (HSV-2) infection is associated with increased susceptibility to HIV infection and control of HSV-2 replication lowers HIV replication [71]. As part of their study investigating the efficacy of curcumin against HIV, Ferreira et al. [10] also examined the effect of curcumin on HSV-2 replication. In primary human GECs, pre-treatment of cells with 5 µM curcumin reduced HSV-2 shedding by 1000-fold, and 50 µM completely prevented production of virus [10]. Investigation of the cellular pathways known to be impacted by curcumin implicated NF-κB was responsible for the effects on HSV-2 [10]. Vitali et al. [72] evaluated the efficacy of curcumin encapsulated by Poly-(Lactic-Co-Glycolic Acid) (PLGA, Cur-PLGA), which can enhance curcumin bioavailability 9-fold [73] against HSV-2 infection in vivo. In mice, although intravaginal (IVAG) delivery of Cur-PLGA (containing 0.5 mg of curcumin) reduced CpG-oligodeoxynucleotides (ODN)-induced acute inflammation, Cur-PLGA had no effect on mice survival following a low- or lethal-dose of HSV-2 [72]. The authors next examined the use of crude curcumin extract in conjunction with polymers that increase curcumin solubility in water [74,75]. Compared to 1 mg of crude curcumin, mice treated with 100 µg of curcumin with PVP-K30 or Soluplus^®^ had increased survival and lower pathology scores [72]. The authors commented that it is difficult to determine if an effective dose is reached when using nanoparticles, whereas it is easier to control the dosage using crude extracts in combination with compounds to improve solubility [72].

### 4.2. Kaposi’s Sarcoma-Associated Herpesvirus

Li et al. [12] noted while reviewing the genes and pathways impacted by curcumin that there was overlap with pathways controlling the redox reaction of apurinic/apyrimidinic endonuclease 1 (APE1), suggesting curcumin could inhibit APE1 redox reactions. Since Kaposi’s sarcoma-associated herpesvirus (KSHV) replication requires the redox function of APE1 [76], the authors investigated whether curcumin could inhibit KSHV replication. Primary effusive lymphoma (PEL) BCBL-1 cells that were latently infected with KSHV were treated with TPA to induce activation of KSHV and subsequently treated with curcumin. Treatment with 30 µM of curcumin effectively blocked reactivation of KSHV by lowering expression of the switch gene replication and transcription activator (RTA), and the delayed-early gene K8 [12]. Curcumin treatment reduced both the intra- and extracellular KSHV genomic DNA levels (IC50 of 8.76 µM and EC50 of 6.68 µM, respectively) [12], indicating that curcumin is an effective treatment for KSHV infection.

### 4.3. Bovine Herpesvirus 1

Bovine herpesvirus 1 (BoHV-1) incubated directly with 10 µM of curcumin caused a significant reduction in viral titer as analyzed by TCID_50_ [23]. However, curcumin did not reduce BoHV-1 titer during a binding assay, suggesting curcumin does not prevent viral attachment but, rather, may be affecting internalization. In Madin-Darby Bovine Kidney (MDBK) cells, 10 µM of curcumin significantly reduced viral titer when applied at the time of infection, but it had no significant effect when added immediately after the one h incubation of cells with the virus inoculum. The authors speculate that curcumin may be inhibiting BoHV-1 infection by upregulating lipid raft formation, a process they previously reported to be disrupted during BoHV-1 infection [23,77]. Reolon et al. [78] evaluated the co-encapsulation of acyclovir, an approved pro-drug that inhibits HSV replication [79], and curcumin into three microparticle (MP) formulations composed of the polymers hydroxypropyl methylcellulose, Eudragit^®^ RS100, or both for efficacy against BoHV-1. In MDBK cells, all three MP formulations significantly reduced plaque formation of BoHV-1 to a greater extent than each antiviral test compound individually at all concentrations (2.7–203.6 µM).

### 4.4. Human Adenovirus

Our group evaluated curcumin against human adenovirus (HAdV). In A549 human lung adenocarcinoma cells infected with HAdV types 4, 5 or 7, curcumin (0–100 µM) caused a dose-dependent decrease in expression of the viral early 1A (E1A) proteins, which are vital for the virus to complete its replicative cycle [80,81], indicating curcumin is effective against multiple types of HAdV. Treatment with curcumin also decreased HAdV-5 viral genome copy numbers, as well as reduced virus recovery as determined by plaque assay [81]. However, the most effective concentrations of curcumin were only slightly lower than the CC_50_ (~68 µM) of curcumin in this cell line, indicating that curcumin may have only a very narrow therapeutic window against HAdV [81].

## 5. Conclusions

Curcumin and its analogues are capable of inhibiting the replication of a diverse group of viruses through numerous mechanisms (summarized in Table 1). However, curcumin has low bioavailability and is rapidly metabolized (reviewed in [82]), compromising curcumin’s effectiveness as an antiviral compound, and likely contributing to the limited success observed in human clinical trials [83]. Additionally, though consumption of curcumin in high doses in humans appears to be safe, numerous studies report in vitro CC_50_ concentrations in tens of micromolars, leading to a relatively low SI, further reducing its potential effectiveness. Nevertheless, research into curcumin formulation technology has shown promise, due to improved solubility, stability and uptake of curcumin and its derivatives [84]. Continued research into such formulations as well as synthesis of novel curcumin-derivates that show greater antiviral activity with reduced toxicity may lead to development of curcumin as a broad-spectrum antiviral for human clinical use.

## Figures and Tables

**Table 1:** Pathways/processes impacted by curcumin and analogues, and their effect on viruses.

Pathway/Process	Antiviral Activity	Virus	References
Actin filament organization	Viral entry	Dengue virus Viral hemorrhagic septicemia virus	[8] [9]
	Replication	Dengue virus	[8]
Anti-inflammation	Replication	Human immunodeficiency virus	[10]
Antioxidation	Replication	Human immunodeficiency virus	[11]
APE1 redox reactions	Replication	Kaposi’s sarcoma-associated herpesvirus	[12]
Cell lipogenesis	Replication	Dengue virus	[8]
Cleavage of eIF4G	Protein expression	Enterovirus 71	[13]
Conformation of viral/cellular surface proteins	Viral attachment	Zika virus Chikungunya virus Vesicular stomatitis virus	[14,15,16]
		Human respiratory syncytial virus	[17,18]
HSC71 expression	Viral entry	Viral hemorrhagic septicemia virus	[9]
NF-κB signalling	Replication	Influenza A virus	[19]
	Viral egress	Herpes simplex virus 2	[10]
PKCδ phosphorylation	Protein expression?	Enterovirus 71	[20]
ROS production	Viricidal	Norovirus	[21,22]
Lipid raft formation		Bovine herpes virus 1	[23]
Viral enzymes	Viral egress Viral protease	Influenza A virus Dengue virus	[24] [8]
Viral proteins	Viral entry	Influenza A virus Porcine reproductive and respiratory syndrome virus	[24] [25]
	Viral protein degradation	Human immunodeficiency virus	[26]
	Viricidal	Norovirus	[21]
